# The Tumor Microenvironment of High Grade Serous Ovarian Cancer

**DOI:** 10.3390/cancers11010021

**Published:** 2018-12-26

**Authors:** M. Sharon Stack, Kenneth P. Nephew, Joanna E. Burdette, Anirban K. Mitra

**Affiliations:** 1 Department of Chemistry and Biochemistry, Harper Cancer Research Institute, University of Notre Dame, Notre Dame, IN 46617, USA; Sharon.Stack.11@nd.edu; 2 Harper Cancer Research Institute, A200 Harper Hall, University of Notre Dame, South Bend, IN 46617, USA; 3 Cell, Molecular and Cancer Biology Program, Medical Sciences, Indiana University School of Medicine, Bloomington, IN 47405, USA; 4 Department of Cellular and Integrative Physiology and Obstetrics and Gynecology, Indiana University School of Medicine, Indianapolis, IN 46202, USA; 5 Indiana University Simon Cancer Center, Indianapolis, IN 46202, USA; 6 Department of Medicinal Chemistry and Pharmacognosy, University of Illinois, Chicago, IL 60607, USA; joannab@uic.edu; 7 Medical Sciences Program, Indiana University School of Medicine, Bloomington, IN 47401, USA; anmitra@indiana.edu; 8 Indiana University Melvin and Bren Simon Cancer Center, Indianapolis, IN 46202, USA; 9 Department of Medical and Molecular Genetics, Indiana University School of Medicine, Indianapolis, IN 46202, USA

The Special Issue on high grade serous ovarian cancer (HGSOC) and the contribution of the tumor micro-environment (TME) consisted of reviews contributed by leaders in the ovarian cancer (OC) field. As HGSOC metastases have a highly complex TME, there is an urgent need to better understand the TME in general, its distinct components in particular, and the role of the TME in the context of disease recurrence and development of chemoresistance. The Special Issue integrated the current understanding of the TME components, including malignant cells, surrounding host stromal cells, and infiltrating (recruited) immune cells. In addition to cellular contributors to the TME, the role of ascites fluid components including soluble factors such as cytokines, chemokines, and growth factors; cell–cell and cell–matrix adhesion molecules; extracellular matrix remodeling; and abnormal vascular and lymphatic networks were the subject of reviews. Reviews covered the relationship between the molecular mechanisms of HGSOC progression, including genomic, epigenomic, and transcriptomic changes, and alterations of the immune cell landscape, as these may provide attractive new molecular targets for HGSOC therapy.

Prof. Dr. Stack et al. [1] illustrated how the aging process has been shown to modulate the TME in ways that are beneficial to the spread and survival of ovarian cancer. Aging hosts have been shown to better facilitate cancer associated inflammation, invasion, and adhesion of cancer cells, while also putting forth a weakened immune response. More research into the unique features of an aging peritoneum is needed to better treat aging HGSOC patients. 

Prof. Dr. Nephew and Prof. Dr. Klemenko illustrated how cells constituting the TME are also involved in epigenetic crosstalk with ovarian cancer cells [2]. Ovarian cancer cells have been shown to epigenetically reprogram a wide variety of cell types in their microenvironment to promote tumor growth, survival, and metastasis. There is also growing evidence to suggest that cells from the tumor microenvironment are capable of epigenetically modifying cancer cells. Prof. Dr. Mitra et al. discussed cancer associated fibroblasts; fibroblasts that have been reprogrammed by cancer cells to support tumor growth, survival, and spread, through the secretion of cancer promoting factors. Further research into their origin and identifying markers is needed in order to better characterize their function within the TME [3]. 

There is still a great deal of research to be done regarding the roles of individual proteins in ovarian cancer. As illustrated by Prof. Dr. Burdette et al. [4], the paired box protein PAX8 is overexpressed in HGSOCs and confers advantages in growth, survival, and migration. While PAX2 has been shown to impart similar growth advantages, expression of it is lost early on in carcinogenesis. Prof. Dr. Hilliard’s review focused on mesothelin which is believed to play a role in survival, proliferation, tumor progression, and adherence. Though its native biological function is poorly understood, it is known to bind to the ovarian cancer biomarker CA125, through which it plays a role in metastasis [5]. Prof. Dr. Hudson and colleagues discussed how many of the signaling pathways implicated in HGSOC converge on the small GTPase Rac1, which is associated not only with actin remodeling, adhesion, and migration, but also EMT, stemness, angiogenesis, and chemoresistance [6]. Rac1’s role in such a high number of cancer associated signaling pathways makes it an appealing target for anticancer therapies.

HGSOC presents unique challenges in the development of effective immunotherapies to combat spread and progression. Prof. Dr. Vanderhyden et al. [7,8] showed how the low mutational burden, recruitment of T-regs, upregulation of immune checkpoint proteins, and heterogeneity associated with epithelial ovarian cancer have served as road blocks in the development of ovarian cancer immune therapies. Prof. Dr. Drakes and Prof. Dr. Stiff illustrated the importance of understanding the factors in the TME that contribute to the immunogenicity of HGSOC in the development of immune therapies and more accurate prognosis of patients [9]. Improvements in immune therapies that result from better characterizing immune modulating TME factors, combined with treatments targeting other areas of the malignancy are important efforts to increase the survival of patients. In their chapter, Prof. Dr. Khabele et al. [10,11] illustrated how macrophages in the TME represent cancer promoting and antitumor forces in ovarian cancer. Cancer promoting M2 tumor associated macrophages (TAM) represent an attractive target for anticancer therapies. Reprogramming of these M2 cells to M1 tumoricidal macrophages constitutes a promising means of manipulating the TME to be less amenable to the malignancy.

HGSOC is a malignancy once thought to originate exclusively from the ovarian surface epithelium. Prof. Dr. Kim et al. [12] reviewed current evidence that suggests HGSOC likely also originates from serous tubal intraepithelial carcinomas (STICs) from the fallopian tube epithelium. Though STIC has been shown to correlate with an increased risk of HGSOC, it is still important to show causation. Additionally, it is important to elucidate the differences between STIC lesions likely to remain benign vs those that are likely to develop into HGSOC.

Epithelial ovarian cancer is commonly associated with metastasis to the peritoneum, though it has been shown to colonize a wide range of other tissues. Prof. Dr. Barbolina’s review focused on the mechanisms of transcoelomic, hematogenous, and lymphatic metastasis [13]. Though most patients typically succumb to transcoelomic, the presence of distant metastasis is associated with worse prognosis. As treatment of transcoelomic metastasis improves, it is likely that more research will have to be devoted to hematogenous and lymphatic spread in order to further improve patient outcomes.

Understanding the interactions between ovarian cancer and metabolites is critical to understanding and treating the disease. In his review, Prof. Dr. Xu outlined how supportive cells have been shown to produce cancer-promoting oncolipids [14]. Improvements to existing detection methods will be valuable in the use of oncolipids as a diagnostic marker in gynecological cancers. Additionally, HGSOC exhibits a reliance on oxidative phosphorylation for its energy needs, making inhibition of the OXPHOS pathway an intriguing target for novel therapies. Prof. Dr. Patankar et al. [15] discussed how OXPHOS inhibition slows proliferation through energy depletion and increases oxidative damage, and through it, cell death. Development of targeted delivery systems for inhibitors of this pathway are needed.

Given the complex roles that the TME plays in supporting tumors, it follows that more sophisticated in vitro models are needed to recapitulate the conditions in which cancer cells exist. Prof. Dr. Kenny et al. [16] described some of the recent developments in 3D modeling of ovarian cancer. Models approximating in situ carcinoma in the fallopian tube, dissemination into the peritoneal cavity, early metastatic attachment to the mesothelial-lined surfaces of the omentum, bowel, and abdominal wall, and late chemoresistant metastases are needed.

The TME of ovarian cancer has numerous unique features that need to be considered in the study and treatment of the disease. According to Prof. Dr. O’Hagan and colleagues [17], recent studies have shown an association between inflammation and an increase in ovarian cancer risk. Though this phenomenon has been well characterized in colon and pancreatic cancers, the mechanisms through which inflammation contributes to ovarian cancer risk need further study. Prof. Dr. Hawkins et al. [18] explained how endometriosis increases the risk of developing endometrioid, clear cell carcinoma, and low grade serous ovarian cancers. The unique tumor microenvironment created by endometriosis facilitates tumorigenesis through upregulation of many gene products associated with ovarian cancers. 

A number of other therapeutic avenues are being explored in ovarian cancer treatment. Efficient targeting of a chemoresistant sub-population of cancer cells known as cancer stem cells (CSC) is a rapidly growing field in the study of high grade serous ovarian cancer. As shown by Prof. Dr. Dahl and Prof. Dr. Roy, the PI3K/PTEN/AKT, Jak/STAT, NFkB, Notch, Wnt, and Hedgehog pathways have all been implicated in the maintenance of cancer stem cells, many of which have therapeutics targeting them currently undergoing clinical trials. Further study is needed to better identify cancer stem cell populations and the mechanisms through which these pathways are involved in their maintenance. 

Another treatment option, known as heated intraperitoneal chemotherapy was discussed by Prof. Dr. Jewell et al. [19] The higher drug levels delivered through HIPEC combined with hyperthermia are thought to increase the efficacy of chemotherapy. Studies showing the benefit of HIPEC along with CRS have been promising, however, use of HIPEC in recurrent cancers warrants further study. The relative safety of this treatment also warrants further investigation. Strategies for treatments targeting supportive components of the TME outlined by Prof. Dr. Matei et al. [20] represent a promising avenue in improving clinical outcomes in patients. Inhibition of angiogenesis, immune therapies, and therapies targeting supportive stromal cells are being explored as therapies to improve survival in HGSOC patients, though more research is needed to increase the efficacy of these approaches.

The planning of this special issue was motivated by the Albert Trust Midwest Ovarian Cancer Coalition (MWOCC) biannual meeting, held in June 2018. We would like to express our deepest gratitude to the conference sponsor, The Leo and Anne Albert Charitable Trust, Mr. Gene Pranzo (Trustee), and Susan Brogan (organizer). We sincerely thank all the attendees and participants who kindly contributed manuscripts of the highest quality to this issue.
